# Novel Self-Healing Metallocopolymers with Pendent 4-Phenyl-2,2′:6′,2″-terpyridine Ligand: Kinetic Studies and Mechanical Properties

**DOI:** 10.3390/polym13111760

**Published:** 2021-05-27

**Authors:** Rose K. Baimuratova, Gulzhian I. Dzhardimalieva, Evgeniy V. Vaganov, Valentina A. Lesnichaya, Gulsara D. Kugabaeva, Kamila A. Kydralieva, Vladimir A. Zhinzhilo, Igor E. Uflyand

**Affiliations:** 1 Institute of Problems of Chemical Physics, Russian Academy of Sciences, Chernogolovka, 142432 Moscow, Russia; rozab@icp.ac.ru (R.K.B.); vaganov@icp.ac.ru (E.V.V.); lesnich@icp.ac.ru (V.A.L.); gulsara_kugabaev@mail.ru (G.D.K.); 2 Moscow Aviation Institute, National Research University, 125993 Moscow, Russia; KydralievaKA@mai.ru; 3 Department of Chemistry, Southern Federal University, 344090 Rostov-on-Don, Russia; i06993@yandex.ru (V.A.Z.); ieuflyand@sfedu.ru (I.E.U.)

**Keywords:** self-healing, metal-containing monomers, terpyridine containing polymers, hydrogel

## Abstract

We report here our successful attempt to obtain self-healing supramolecular hydrogels with new metal-containing monomers (MCMs) with pendent 4-phenyl-2,2′:6′,2″-terpyridine metal complexes as reversible moieties by free radical copolymerization of MCMs with vinyl monomers, such as acrylic acid and acrylamide. The resulting metal-polymer hydrogels demonstrate a developed system of hydrogen, coordination and electron-complementary π–π stacking interactions, which play a critical role in achieving self-healing. Kinetic data show that the addition of a third metal-containing comonomer to the system decreases the initial polymerization rate, which is due to the specific effect of the metal group located in close proximity of the active center on the growth of radicals.

## 1. Introduction

**Scheme 1:**
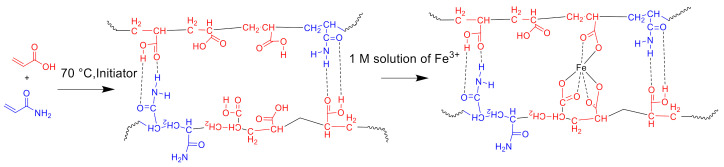
Scheme for the synthesis of the Poly(AA-co-AM) network with subsequent crosslinking of polymer chains with Fe^3+^ions.

Self-healing polymers (SHPs) are one of the most important classes of materials discovered in the 20th century, and they can be characterized as systems capable of healing local mechanical damage, such as cracks or scratches [1,2]. Typically, self-healing materials should be classified as non-autonomous, stimulable, and autonomous by the fact of assistance (e.g., light, heat, electricity, pH, etc.), which may or may not be required, respectively, to start the healing process [3,4]. In the scientific literature, there are also definitions such as extrinsic and intrinsic self-healing systems. The first involves the introduction of additional healing agents into the polymer matrix (for example, by encapsulation), but detection of cracks and activation of repair are impossible without outside help [5]. In contrast, intrinsic SHPs contain physical, supramolecular interactions, or specific chemical bonds in their own structure, the change in the configuration of which allows easy/quick recover after damage, leading to a faster self-healing process [6]. Such systems usually have reversible covalent bonds based on the Diels–Alder reaction [7,8], disulfide bonds [9], [2+2] cycloadditions [10], or non-covalent interactions, such as π–π interactions [11], hydrogen bonds [12], host-guest interactions [13], ionic interactions [14], metal-ligand (M-L) interactions [15], and/or their orthogonal combinations [16]. Among them, the inclusion of specific flexible or even switchable M–L bonds in a material is an attractive strategy for creating polymer networks with self-healing and functional properties, such as electrical conductivity [17], luminescence [18], or thermo- [19] and light-response [20]. Therefore, over the past 10 years, various types of self-healing polymers with ionic groups (ionomers) [21,22], double-network hydrogels [23,24] with different metal salts (M = Ni, Cu, Fe, Rh, Ag, etc.), and N-heterocyclic carbenes [25] have been intensively studied [26,27]. Heterocyclic pyridine-containing compounds are the most promising ligands for reversibly assembled and disassembled metal ion-ligand interactions [28,29]. Nitrogen-based multidentate aromatic ligands, such as terpyridine, are also often used to prepare self-healing polymers with functional properties due to their ability to bind different amounts of metal ions [30,31]. S. Kupfer et al. [32] suggested that thermally induced self-healing mechanism of such systems is based on the reversible thermal decomplexation of the bisterpyridine metal complexes. It should be noted that the fastest self-healing properties have been established in supramolecularly assembled metallogels based on terpyridine-functionalized polynorbornenes with Zn^2+^ ions, due to the lower affinity of metal-ligand binding [33]. Although many synthetic approaches have been used in order to attach metal-containing functional groups to the reactant’s material, such as polymer-analogous transformations [34], polycondensation of metal-containing precursors, when metal ion is incorporated into backbone and its elimination results in the destruction of polymer [35], (co)polymerization of metal-containing monomers (MCM) is most attractive convenient one-stage method for synthesis of metal-containing polymers [36]. However, as far as we know, this approach has not been used for the synthesis of self-healing polymers containing metal complexes. Various chemical two-step strategies have been discovered. The first stage is the general procedure for the synthesis of supramolecular systems, such as ionic and free radical (co)polymerization of vinyl monomers (for example, using 4′-vinylterpyridine monomer in the case of the introduction of terpyridine moieties into the polymer backbone [37]) or reversible addition-fragmentation chain-transfer polymerization [38], and more recently, ring-opening metathesis polymerization [33] with subsequent crosslinking of polymer chains using a solution of salts of various metals (Scheme 1) [39].

We report here our successful attempt to obtain self-healing supramolecular hydrogels bearing terpyridine-metal complexes by free radical copolymerization of MCMs with vinyl monomers, such as acrylic acid (AA) and acrylamide (AM). The main goal of the study is to develop new metal-containing copolymers with pendent 4-phenyl-2,2′:6′,2″-terpyridine-containing metal complexes as reversible moieties for the formation of self-healing metallopolymer networks. Particular attention is paid to the study of the kinetics of copolymerization of MCMs.

## 2. Materials and Methods

### 2.1. Starting Materials

**Scheme 2:**
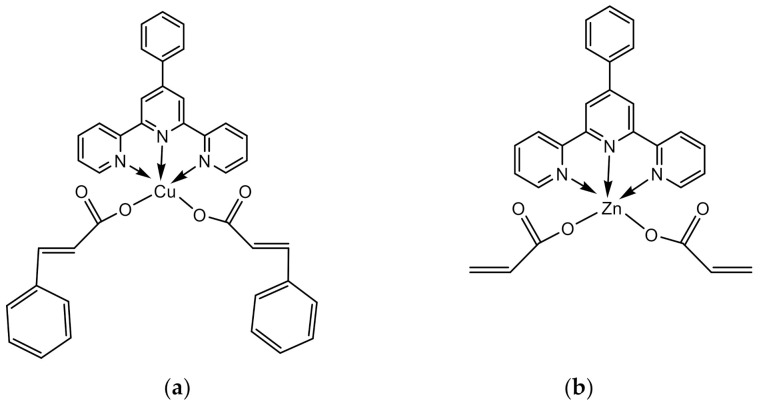
Schematic representation of synthesized MCMs with pendent 4-phenyl-2,2′:6′,2″-terpyridine ligand ((**a**)—CuCinnamatePhTpy (MCM1), (**b**)—ZnAcrylatePhTpy (MCM2)).

Acrylamide (AM, ≥99%) and potassium persulfate (KPS, ≥99.0%) was purchased from Aldrich (Moscow, Russia) and used without further purification. The stabilized acrylic acid, obtained from Sigma-Aldrich (Moscow, Russia) was purified according to standard procedures by vacuum distillation. The purity of the initial AA was monitored by liquid adsorption chromatography. The synthesis and characteristics of new bifunctional copper and zinc containing monomers based on unsaturated acids (cinnamic and acrylic, respectively) and 4-phenyl-2,2′:6′,2″-terpyridine ligand were described earlier [40]. A schematic representation of the synthesized MCMs with pendent 4-phenyl-2,2′:6′,2″-terpyridine ligand is shown in Scheme 2. Water used as a solvent in all the experiments was distilled, deionized and deaerated by bubbling argon gas through it.

### 2.2. Analytical Methods

#### 2.2.1. Characterization of the Structure and Composition of the Polymers

The content of C, H, N was determined on an element analyzer “Vario Micro cube” (Elementar GmbH, Hanau, Germany). The content of metals (Cu, Zn) was found using an atomic absorption spectrometer “AAS-3” (Zeiss, Jena, Germany). DSC and TGA curves and mass spectra of gaseous products were recorded using an STA 409 C Luxx (NETZSCH, Selb, Germany) conjugated with quadrupole mass spectrometer QMS 403C Aeolos and METZSCH STA 409 PC/PG. The samples were heated in an argon atmosphere at a heating rate of 10 K min^−1^ in the temperature range 30–500 °C. Attenuated total reflection infrared (ATR-FTIR) spectra of the polymers were recorded on an ALPHA FTIR spectrometer (Bruker, Shelton, CT, USA). A monolithic diamond crystal with an aperture angle of 45° was used as an element of internal reflection. The wavenumber was varied from 4000 to 1000 cm^−1^; number of scans—16; resolution is 4 cm^−1^. UV-vis-NIR-spectra were recorded using a SPECS-SSP-705-1 spectrometer (JSC “Spectroscopic Systems”, Moscow, Russia). The obtained copolymers were analyzed for phase identification and crystallinity by X-ray powder diffraction (XRD) on an ARLX’TRA diffractometer (Thermo Electron Corp. Waltham, MA, USA) (step size—0.02 °C, radiation—CuKα, λ = 1.540598 Å).

#### 2.2.2. Study of Kinetics of Copolymerization

The kinetics of radical copolymerization was studied on a differential automatic microcalorimeter (DAK-1-1, Experimental Plant of Scientific Instrumentation NTO, Chernogolovka, Russia) in the mode of direct recording of the rate of heat release (dQ/dt) under isothermal conditions (at 60 and 80 °C). The reaction mixtures used for microcalorimetric studies were placed in ampoules, carefully evacuated to a pressure of no higher than 10^−4^ Pa, and sealed. In kinetic calculations, the value of ΔH for AM and AA polymerization was taken equal to 77.3 and 81.5 kJ/mol, respectively [41]. The concentration of acrylate and acrylamide bonds significantly exceeds the concentration of bonds of MCM by three orders of magnitude, which made it possible to neglect the difference in the thermal effects of polymerization of cinnamate groups when calculating the conversion of double bonds.

#### 2.2.3. Mechanical Properties

Tensile stress–strain measurements were carried out on a universal testing machine Zwick/Roel TC-FR010 in accordance with standards at a strain rate of 1 mm/min in air at room temperature. The samples were cut from gel sheets onto dogbone shape samples with an initial gauge length of 50 mm (L_0_) and a width of 1 mm (Figure S1). Tests were performed on a series of at least 5 samples for each copolymer to ensure repeatability. The nominal stress (σ) and strain (ε) were recorded as the applied load divided by the original cross-sectional area of the samples and the clamp displacement divided by L_0_. Young’s modulus (E) was obtained from the initial slope of the stress–strain curve at a strain of less than 1%.

#### 2.2.4. Self-Healing Properties

Two groups of experiments to measure self-healing properties were carried out simultaneously at room temperature. In the first series of tests, a hydrogel sample was cut into two separate pieces, and the cut surfaces of the samples were manually joined so that the cut surfaces could be in sufficient contact with each other without external stimuli in a sealed container to minimize water evaporation. The healing process was monitored visually using tweezers to observe the healing state. The hydrogels showing the fastest self-healing (less than 48 h) were selected to evaluate healing efficiency by comparing the tensile strength of the healed specimen (σ _h_) with the original healed specimen (σ_o_) or E_h_ and E_o_-modulus of elasticity of the original and healed samples, respectively. The healing efficiency (Z) can be calculated using the followed Equations (1) and (2):

(1) $$Z_{\mathrm{stress}}=\frac{\sigma_{h}}{\sigma_{0}}\times 100\%$$

(2) $$Z_{E}=\frac{E_{h}}{E_{0}}\times 100\%$$

In addition, it has been shown that it is possible to accelerate the healing process (up to less than 30 min) with an acidic solution, for example with 0.1 M HCl solution. It is known that terminalcarboxyl groups are fully protonated at low pH which allows them to form hydrogen bonding with amide groups or other terminal carboxyl groups [42,43].

### 2.3. Synthesis of Copolymers

Copolymers P(AM-co-AA-co-MCM) and P(AM-co-AA) were obtained by free radical polymerization of a monomer–solvent mixture consisting of a given amount of initiator (KPS), vinyl monomers (AA, AM) and a small amount of MCM (MCM = CuCinnamatePhTpy (MCM1) or ZnAcrylatePhTpy (MCM2)) at 60 or 80 °C for 6 h. The concentration of the components and the AA/AM/MCM ratio for the synthesis of copolymers of various compositions were adjusted and are shown in Table 1. The reaction mixture was placed in an ampoule and degassed three times until a residual pressure of 5 × 10^−3^ Pa was reached. The resulting product was a highly viscous liquid from light turquoise to green in the case of copper-containing polymers, from white to light yellow for zinc-containing polymers, and transparent for binary copolymers without the addition of a third metal-containing comonomer (Figure S2). The copolymer was separated from unreacted residual monomers by precipitation in acetone and dried for use until a constant weight was observed. The solid copolymers were redissolved in water with stirring and low heating until homogeneous and clear aqueous solutions were obtained, which could be used for further casting.

## 3. Results and Discussion

### 3.1. Characterization of the Structure and Composition of the Polymers

The solid copolymers P(AM-co-AA-co-MCM), which were separated from unreacted residual monomers by precipitation in acetone, were analyzed for C, H, N, and metal content (Table 2). The calculated and determined content of elements is different. This is due to the possibility of preserving physically coordinated water molecules in polymer networks, as well as the relative activity of monomers, mainly determined by the peculiarities of their chemical structure.

**Figure 1:**
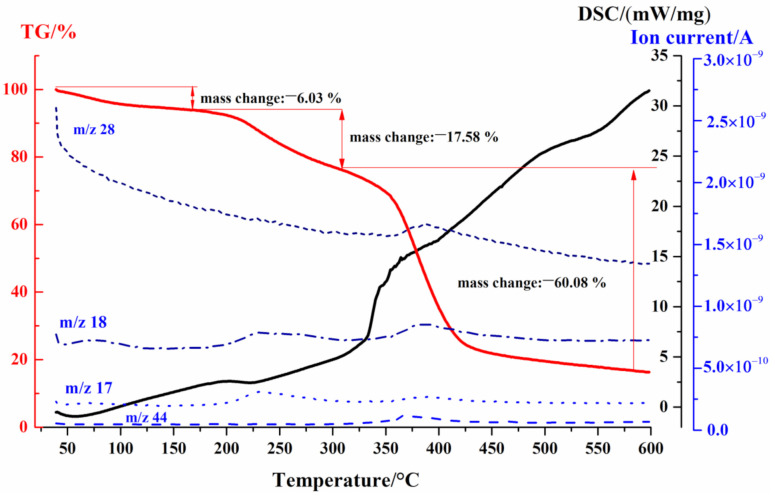
DSC and TGA curves of copolymer isolated from Gel 6 [AM/AA/MCM1 = 84.5/14.25/1.25 (%/%/%)].

According to the TGA and DSC curves (Figure 1; Table 3; Figures S3–S5), the resulting copolymer decomposes in at least three stages. The first stage takes place at a temperature of 40 to 168 °C with a maximum decomposition rate at 68 °C and a weight loss of 6.03 wt.%. The second stage starts at 168 °C and ends at 308 °C with a maximum decomposition rate at 227 °C and a weight loss of 17.58 wt.%. Then the main stage of decomposition is observed in the range of 308–450 °C with maximum decomposition at 380 °C and a total weight loss of 60.08 wt.%. According to ion fragmentation mass spectrometry, the first and second stages of decomposition of copolymers P(AM-co-AA-co-MCM) are mainly accompanied by the release of water (*m*/*z* = 18;17). The third step observed at temperatures above 310 °C, is associated with decarboxylation (*m*/*z* = 28; 44) and decomposition of the polymer backbone into acrylamide fragments CH_3_CN (*m*/*z* = 48), C_2_H_3_CN (*m*/*z* = 60) C_3_H_5_CN (*m*/*z* = 74), C_2_H_3_CONH_2_ (*m*/*z* = 78), and acrylate fragments CH_3_CHO (*m*/*z* = 51). Characteristic peaks of benzene derivatives with *m*/*z* 78–76, 113, 127, fragment ions with *m*/*z* 56.99, indicating the removal of 4′-phenyl-2,2′:6′,2′-terpyridine and cinnamic acid fragments by deeper decomposition of the polymer chain are observed at temperatures above 350 °C in the mass spectrum.

**Figure 2:**
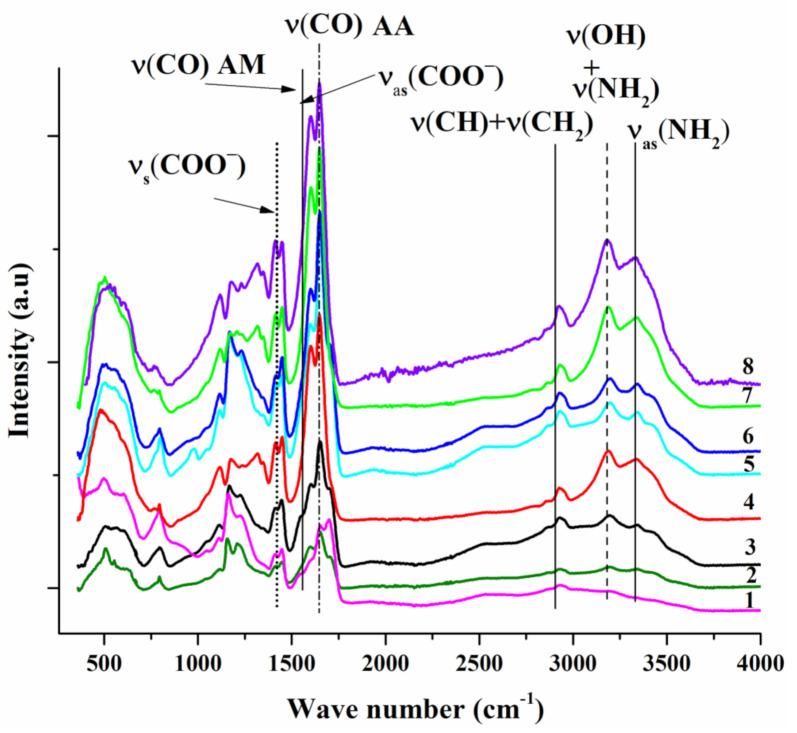
IR spectra of copolymers isolated from gel solutions by precipitation in acetone and dried in air: 1—[AM/AA/MCM1 = 14.5/84.5/1]; 2—[AM/AA = 50/50] 50% sol.; 3—[AM/AA/MCM2 = 49.5/49.5/1] 50% sol.; 4—[AM/AA/MCM1 = 84.5/14.5/1]; 5—[AM/AA/MCM1 = 49.5/49.5/1] 50% sol.; 6—[AM/AA/MCM1 = 49.5/49.5/1]; 7—[AM/AA/MCM2 = 84.5/14.5/1]; 8—[AM/AA/MCM1 = 84.5/14.25/1.25].

**Scheme 3:**
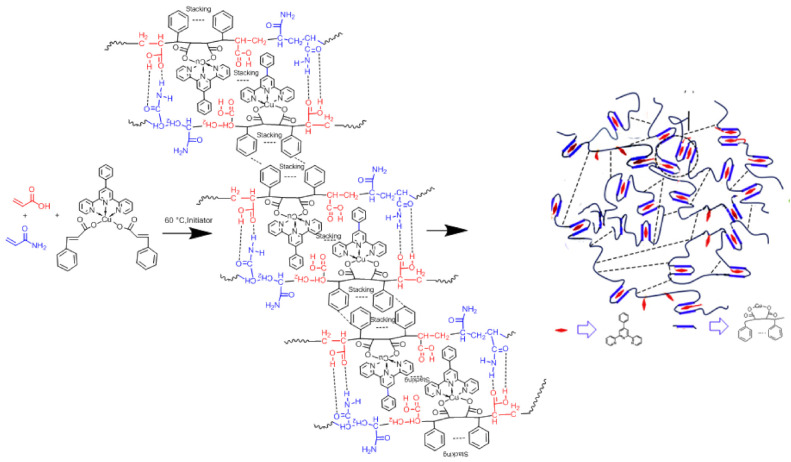
Schematic representation of the structure of the obtained copolymer P(AM-co-AA-co- MCM1), demonstrating a developed system of hydrogen, coordination and electron-complementary π–π stacking interactions, which play a decisive role in achieving self-healing.

According to IR data (Figure 2), all synthesized copolymers show a strong broad peak in the range of 3500 – 2800 cm^−1^, indicating the presence of intermolecular hydrogen bonds in the polymer. The peak is observed at 3340 cm^−1^, related to the stretching vibration of the NH group of acrylamide molecules, and is overlapped by the stretching vibration of the OH groups of acrylate moieties. The peak located at 2928 cm^−1^ can be attributed to the C–H stretching of the methyl and methylene groups of the polymer backbone. It should be noted that the interaction of the amide group (δ_NH_+ν_CN_) and ν_C = O_ in the carboxyl group leads to a shift of the C=O band (1648 cm^−1^), indicating that strong hydrogen bonds are formed between –COOH and –CONH_2_, as shown in Scheme 3 [23,44]. The peaks arising at 1449–1415 cm^−1^, 1600–1648 cm^−1^, and 531 cm^−1^ can be caused by asymmetric and symmetric stretching vibrations of COO groups and stretching vibrations of M–O bonds of metal-containing units in the polymer, respectively.

**Figure 3:**
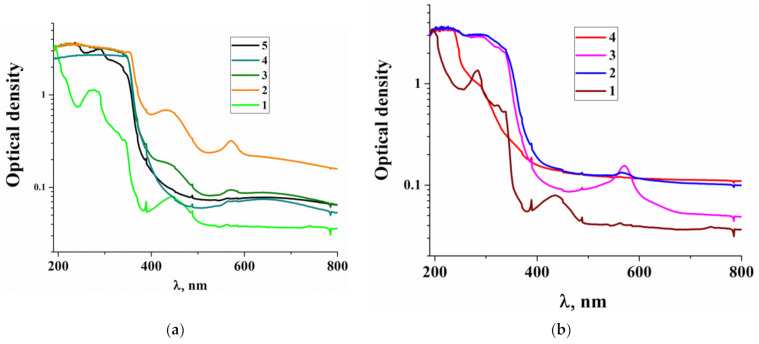
UV spectra of the obtained copolymers based on vinyl monomers such as AA, AM and MCM (MCM = MCM1 (**a**) or MCM2 (**b**)): (**a**) 1—MCM1 in ethanol; 2—[AM/AA/MCM1 = 49.5/49.5/1] 50% sol.; 3—[AM/AA/MCM1 = 49.5/49.5/1] 80% sol.; 4—[AM/AA/MCM1 = 14.5/84.5/1]; 5—[AM/AA/MCM1 = 84.5/14.5/1]; (**b**) 1—MCM2 in ethanol; 2—[AM/AA/MCM2 = 84.5/14.5/1]; 3—[AM/AA/MCM2 = 49.5/49.5/1]; 4—[AM/[AC = 50/50].

Characterization of copolymers and complexes using UV-vis spectroscopy is shown on Figure 3. According to the absorption spectra of complexes dissolved in ethanol, sharp absorption bands at 235–290 nm, characteristic of PhTpy ligands [45], are observed at 250–300 nm for CuCinnamatePhTpy and at 260–300 nm for ZnAcrylatePhTpy, respectively. Metal-to-ligand charge transfer (MLCT) band of zinc(II) and copper(II) complexes observed at 435 and at 446 nm indicates successful complexation and shows a bathochromic shift up to 572 and 574 nm for polymers with an equivalent number of AA and AM units.

**Figure 4:**
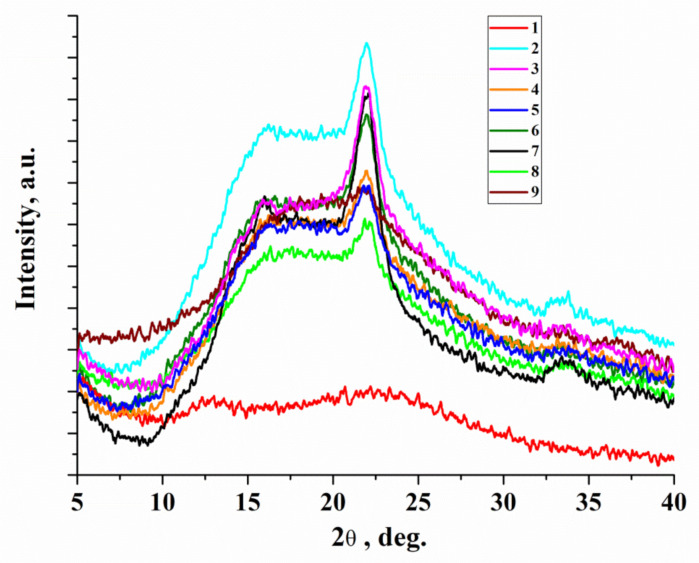
XRD patterns of copolymers P(AM-co-AA-co-MCM) when MCM = MCM1 or MCM2: 1—[AM/AA = 85/15]; 2—[AM/AA/MCM2 = 84.5/14.25/1.25]; 3—[AM/AA/MCM1 = 84.5/14.5/1]; 4—[AM/AA/MCM2 = 49.5/49.5/1]; 5—[AM/AA/MCM1 = 49.5/49.5/1]; 6—[AM/AA/MCM2 = 14.5/84.5/1]; 7—[AM/AA/MCM1 = 14.5/84.5/1]; 8—[AM/AA/MCM2 = 49.5/49.5/1] in 50% sol.; 9—[AM/AA/MCM1 = 49.5/49.5/1] in 50% sol.

The addition of MCM leads to the appearance of regions of crystallinity in the resulting supramolecular systems (Figure 4).

Perhaps the appearance of crystallinity is associated with the influence of π–π-stacking interactions between the electron-deficient and electron-rich benzene rings of phenylterpyridine of the supramolecular structure of the polymer. This behavior can be represented by Scheme 3.

### 3.2. Study of Kinetics of Copolymerization

The rate of heat release in the study of kinetic processes on the calorimeter is related to the pulses of the analog-to-digital current converter by the following equation:

(3) $$\frac{\mathrm{dQ}}{\mathrm{dt}}=E\Delta +F\frac{d\Delta }{\mathrm{dt}},$$

where Δ is the magnitude of the impulse, arb. units; E and F are the constants of the calorimeter determined during the calibration of the instrument, J/a.u.s. and J/a.u., respectively.

The kinetic parameters of polymerization were calculated from the obtained calorimetric curves dQ/dt = f(t) according to the standard procedure:

(a) the polymerization rate is reduced to the initial concentration of double bonds

(4) $$\frac{W}{{\left[M\right]}_{0}}=\frac{\mathrm{dQ}}{\mathrm{dt}}\frac{\mathrm{MM}}{Q_{\mathrm{sp}}n\omega m},$$

where m is the mass of the reaction mixture placed in the calorimeter, g; ω is the mass fraction of the monomer in the reaction mixture; MM is the molecular weight of the monomer, g/mol; n is the number of double bonds in the monomer molecule; Q_sp_ is the specific heat of polymerization upon opening one double bond of the monomer, J/mol.

(b) conversion of double bonds

(5) $$C_{i}=\frac{W}{{\left[M\right]}_{0}}\mathrm{dt}+C_{i-1},$$

where C_i_ and C_i-1_ are the currently calculated conversions of double bonds and the conversion of double bonds at the moment of time preceding this by the interval dt.

(c) the polymerization rate is reduced to the current concentration of double bonds

(6) $$\frac{W}{\left[M\right]}=\frac{W}{{\left[M\right]}_{0}}\frac{1}{1-C}.$$

**Figure 5:**
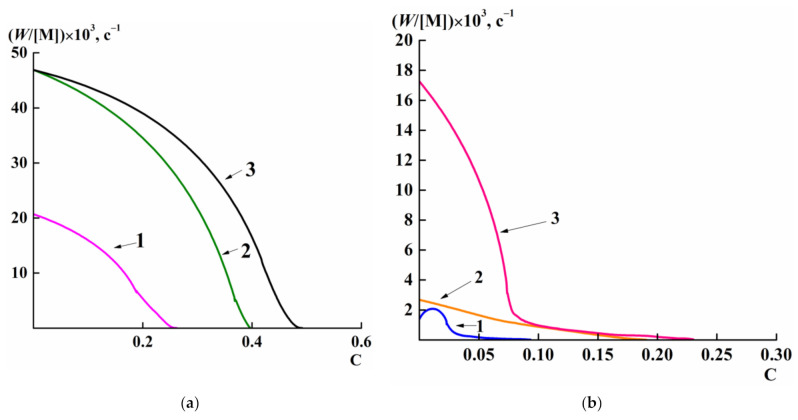
Effect of the AA/AM ratio on the conversion of С=С groups in the system with MCM1 (**a**) and MCM2 (**b**): (**а**) 1—[AM/AA/MCM1 = 84.5/14.5/1]; 2—[AM/AA/MCM1 = 49.5/49.5/1]; 3—[AM/AA/MCM1 = 14.5/84.5/1] (60 °С, [KPS] = 0.2, [Н_2_О] = 80 wt.%); (**b**) 1—[AM/AA/MCM2 = 14.5/84.5/1]; 2—[AM/AA/MCM2 = 84.5/14.5/1]; 3—[AM/AA/MCM2 = 49.5/49.5/1]; (80 °С, [KPS] = 0.2, [Н_2_О] = 80 wt.%).

Kinetic data show that the addition of a third metal-containing comonomer to the system always decreases the initial polymerization rate and the conversion of С=С groups in the reaction. Moreover, the addition of a zinc-containing monomer decreases several times more than a copper-containing monomer (Figure 5).

**Figure 6:**
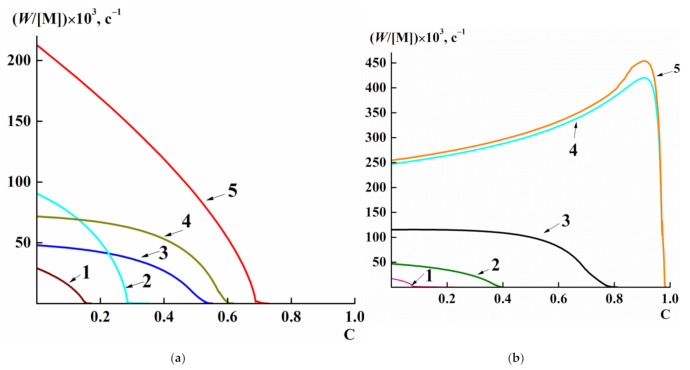
(**a**) Influence of the AA/AM ratio and temperature on the conversion of С=С groups in P(AM-co-AA): 1—[AM/AA = 85/15] at 60 °C; 2—[AM/AA = 85/15] at 80 °C; 3—[AM/AA = 15/85] at 60 °C; 4—[AM/AA = 15/85] at 80 °C; 5—[AM/AA = 50/50] at 80 °C ([KPS] = 0.2 wt.%, [Н_2_О] = 80 wt.%); (**b**) Effect of association on the conversion of С=С groups in the systems 1—[AM/AA/MCM2 = 49.5/49.5/1] in 80wt.%; 2—[AM/AA/MCM1 = 49.5/49.5/1] in 80 wt.% sol. at 60 °C; 3—[AM/AA/MCM2 = 49.5/49.5/1] in 50wt.% sol. at 80 °C; 4—[AM/AA/MCM1 = 49.5/49.5/1] in 50 wt.% sol. at 60 °C; 5—[AM/AA = 50/50] in 50 wt.% sol. at 60 °C (AA/AM = 1, [KPS] = 0.2 wt.%).

We have previously observed such effects for many copolymerization reactions with MCMs [46]. The effect of the association on the conversion of С=С groups in the systems with AA/AM = 1 ratio is also observed. Thus, with the same ratio of AA comonomers with AM and MCM1 (Figure 6b), a decrease in the amount of solvent from 80 to 50 wt.% increases the initial rate of polymerization by 5 times. This can be associated with both a change in viscosity during radical polymerization (gel-effect) and with nonspecific solvation by monomer or solvent molecules (including due to hydrogen bonds) of macroradical carriers of the polymer chain with the formation of π-complexes [41]. It should be noted that an increase in the amount of AM in the reaction system not only lowers the initial rate of polymerization, but also affects the conversion of the double bond in the system, which can be observed due to a change in the pH of the medium with varying comonomer ratio or chain transfer reactions (for example, the chain transfer constant to AM can be k_M_ = (0.79–7.8)·10^−5^ [47]). The study of the kinetics MCM copolymerization makes it possible to regulate the spatial and electronic structure of the resulting metal-supramolecular polymers, as well as their functional properties.

### 3.3. Study of Self-Healing and Mechanical Properties

**Figure 7:**
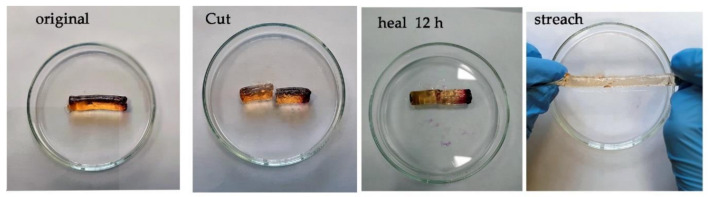
Photographs of the healing process of the gel 13 ([AM/AA/MCM2 = 49.5/49.5/1] in 50 wt.% sol), demonstrating its mechanical deformation and recoverability.

It is known that traditional synthetic polymer hydrogels usually have weak mechanical strength, low impact strength, and low recoverability [48,49]. This is largely due to their unified grid structure, which leads to internal structural heterogeneity, due to the irregular distribution of cross-linking sites and the absence of effective energy dissipation mechanisms. From this point of view, the resulting materials have improved mechanical characteristics as well as better recyclability and self-healing properties. In a number of works, the obtained hydrogels did not dissolve in deionized water, acidic and alkaline solution, which negatively affects the possibility of reuse [50]. Accordingly, a series of repeated self-healing experiments showed that the resulting copolymers of AA, AM and MCM, consisting of a mixed-ligand complex of divalent metals [M = Cu(II), Zn(II)] and unsaturated acids (cinnamic and acrylic) and 4-phenyl-2,2′:6′,2″-terpyridine exhibit self-healing properties (Figure 7; Figures S6 and S7). As shown in Figure 7, the hydrogel with length of ~4 cm was first cut at the center to form a 6 mm width separation. Then the hydrogels were placed in a desiccator at room temperature with a slight wetting with water. It was found that the cut gradually diminished and completely disappeared within 12 h. The hydrogels showing the fastest (less than 48 h) self-healing were chosen to evaluate the healing efficiency by comparing the tensile strength of the healed specimen with the original healed specimen.

**Figure 8:**
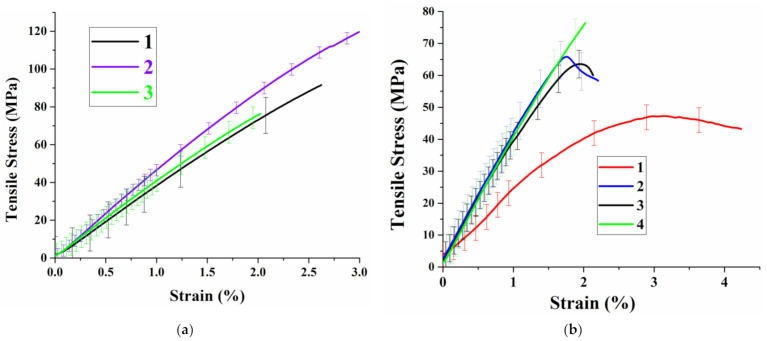
(**a**) Effect of MCM addition on physical and mechanical properties of P(AM-co-AA): 1—[AM/AA = 50/50]; 2—[AM/AA/MCM2 = 49.5/49.5/1]; 3—[AM/AA/MCM1 = 49.5/49.5/1] (AA/AM (mol) = 1) prepared in 80 wt.% sol.; (**b**) Influence of the AM/AA ratio on the mechanical properties of the ternary copolymer P(AM-co-AA-co-MCM1) 1—[AM/AA/MCM1 = 14.5/84.5/1]; 2—[AM/AA/MCM1 = 84.5/14.5/1]; 3—[AM/AA/MCM1 = 49.5/49.5/1] prepared in 80 wt.% sol.); 4—[AM/AA/MCM1 = 49.5/49.5/1] prepared in 50 wt.% sol.).

The stress–strain (σ-ε) curves of dogbone shape samples are shown in Figure 8. The copolymers have shown impressive mechanical properties: tensile strength can reach 110 MPa. It was shown that, at an equimolar ratio of AM and AA, the addition of a zinc-containing monomer to the structure leads to an increase in tensile strength by 30%.

**Figure 9:**
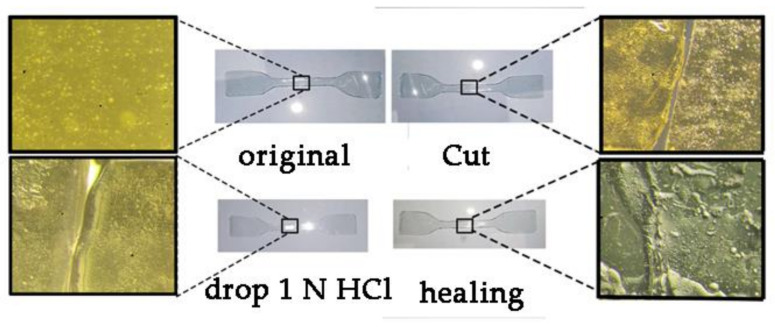
Optical microscope photographs of the healing process of dogbone shape samples of copolymer 8-[AM/AA/MCM1 = 84.5/14.5/1] using an acidic solution (1 drop of 0.1 M HCl solution).

Although the films have good mechanical properties, they can be healed at room temperature with an acidic solution (1 drop of 0.1 M HCl solution), as shown in Figure 9. Thus, finding autonomous self-healing is still a challenge for materials that exhibit simultaneously improved mechanical properties and high self-healing ability.

Supramolecular AM-co-AA-co-MCM hydrogels exhibit the ability to self-repair mainly due to the reversible complexation reaction between metal ions and terpyridine moieties, electrostatic interaction between the carboxyl anion and copper and zinc ions, as well as the use of reversible hydrogen bonds between carboxyl and amide groups, which is schematically illustrated in Scheme 3.

**Figure 10:**
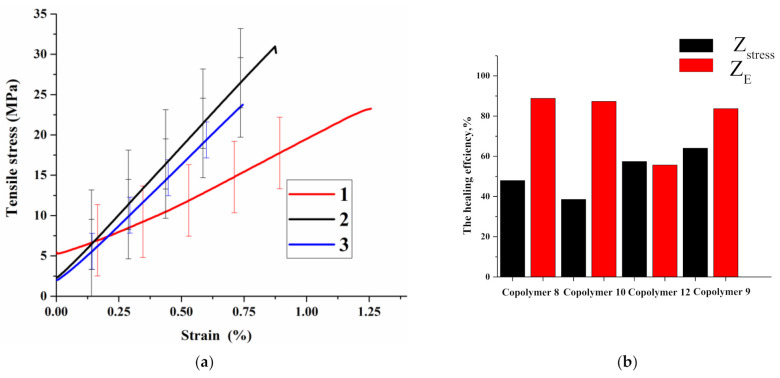
(**a**) Mechanical properties of copolymers after healing for 12 h; (**b**) healing efficiency after 12 h: 1—[AM/AA/MCM1 = 14.5/84.5/1]; 2—[AM/AA/MCM1 = 84.5/14.5/1]; 3—[AM/AA/MCM1 = 49.5/49.5/1].

As shown in Figure 10, the self-healing efficiency of mechanical properties depends on the composition of the copolymer and can reach 78–80%. It has been shown that in copolymers with a high acrylamide content, the healing efficiency is higher. This behavior, apparently, is due to the fact that with an increase in the content of acrylamide in the copolymer there is a weakening of their mechanical properties, as also reported in [51]. The system becomes more dynamic, which in turn leads to an improvement in self-healing properties, as shown by many studies [52].

**Figure 11:**
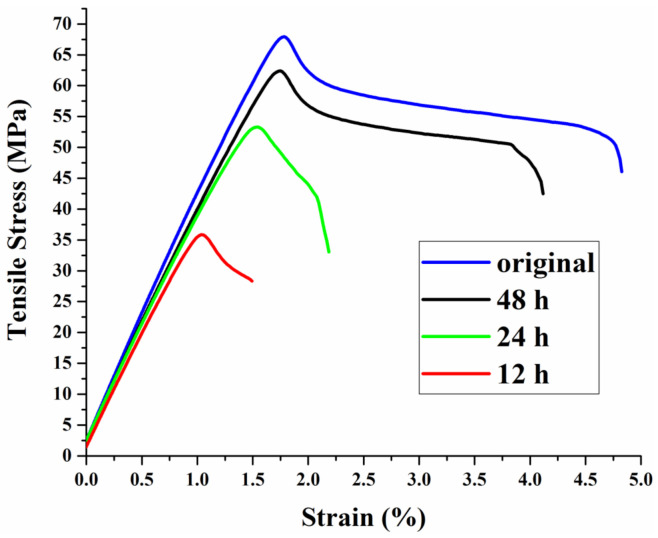
Stress–strain curves of the healing sample (copolymer 8-[AM/AA/MCM1 = 84.5/14.5/1]) after 12, 24, and 48 h.

The recovery property with different waiting times was studied on example of the copolymer 8, where we observed an increase in healing efficiency over time (Figure 11).

## 4. Conclusions

In conclusion, we have designed a new kind of self-healing supramolecular materials with metal-containing monomers (MCMs) with pendent 4-phenyl-2,2′:6′,2″-terpyridine metal complexes as reversible moieties. A new approach was developed through the one-step (co)polymerization of MCMs with vinyl monomers, such as acrylic acid (AA) and acrylamide (AM) to create self-healing polymers. The obtained metal-polymer hydrogels demonstrate a developed system of non-covalent hydrogen, coordination, and electron-complementary π–π stacking interactions, having a significant effect on the ability to self-healing. Kinetic data show that copolymerization of vinyl monomers in the presence of MСM is accompanied by a decrease in the rate of polymerization due to the specific effect of the metal group. Further investigation will be related to the study of the mechanism of self-healing of the obtained metallosupramolecular polymers and influence of the molar fraction of AA and AM, as well as the concentration of MCM on the mechanical properties of the produced self-healing materials.

## Figures and Tables

**Table 1:** Compositions of precursor solutions for the synthesis of copolymers of various compositions.

Concentration (wt.%)							AM/AA/MTpy (%/%/%)
Samples	Water	AA	AM	KPS	MCM1	MCM2	
Gel 1	80	17	3	0.2	-	-	15/85
Gel 2	80	10	10	0.2	-	-	50/50
Gel 3	80	3	17	0.2	-	-	85/15
Gel 4	50	25	25	0.2	-	-	50/50
Gel 5	80	2.85	16.9	0.2	-	0.25	84.5/14.25/1.25
Gel 6	80	2.85	16.9	0.2	0.25	-	84.5/14.25/1.25
Gel 7	80	2.9	16.9	0.2	-	0.2	84.5/14.5/1
Gel 8	80	2.9	16.9	0.2	0.2	-	84.5/14.5/1
Gel 9	80	9.9	9.9	0.2	-	0.2	49.5/49.5/1
Gel 10	80	9.9	9.9	0.2	0.2	-	49.5/49.5/1
Gel 11	80	16.9	2.9	0.2	-	0.2	14.5/84.5/1
Gel 12	80	16.9	2.9	0.2	0.2	-	14.5/84.5/1
Gel 13	50	24.9	24.9	0.2	-	0.2	49.5/49.5/1
Gel 14	50	24.9	24.9	0.2	0.2	-	49.5/49.5/1

**Table 2:** Elemental analysis results for MCM copolymers (MCM1 or MCM2) with vinyl monomers such as AA and AM.

Sample	Elemental content (wt.%) (Found/Calculated)				AM/AA/MCM (%/%/%)	AM/AA/MCM (%/%/%)
	C	H	N	M	Calculated	Found
Copolymer 5	41.77	7.61	13.54	0.28/0.25	84.5/14.25/1.25	59.7/38.9/1.4
Copolymer 6	42.58	7.52	14.27	0.21/0.17	84.5/14.25/1.25	62.3/37.7/1.1
Copolymer 7	40.97	7.46	13.52	0.09/0.2	84.5/14.5/1	63.0/36.3/0.7
Copolymer 8	42.17	7.49	13.50	0.11/0.14	84.5/14.5/1	59.5/39.5/1
Copolymer 9	44.03	6.99	8.65	0.09/0.14	49.5/49.5/1	39.6/59.8/0.6
Copolymer 10	43.83	7.05	8.82	0.15/0.1	49.5/49.5/1	39.6/59.8/0.6

**Table 3:** Thermal properties of solid copolymers P(AM-co-AA-co-MCM).

№	Sample	TGA data				T_g_ (°C) (ASTM)	Residue at 550 °C (wt.%)
		T_5%_ (°C)	T_10%_ (°C)	T_20%_ (°C)	T_max_ (°C)		
1	Copolymer 2	173	202	269	217; 382	83	16.1
2	Copolymer 6	120	218	275	217; 378	88	19.5
3	Copolymer 7	117	185	253	222; 382	89	23.8
4	Copolymer 8	71	120	228	221; 283; 383	89	23.4
5	Copolymer 9	124	177	220	210; 381	78	3.4
6	Copolymer 10	116	175	235	220; 373	80	16.2
7	Copolymer 12	198	262	324	200; 278; 379	68	21.3
8	Copolymer 14	177	208	330	180; 382	67	33.6

T_5%,_ T_10%,_ T_20%_—temperature of 5, 10, and 20% weight loss, respectively; T_max_—temperature of the maximum decomposition rate from DTG thermogram; T_g_—glass transition temperature.

## Data Availability

Not applicable.

## Supplementary Materials

The following are available online https://www.mdpi.com/article/10.3390/polym13111760/s1, Figure S1: Gel sheet (a) and the samples for the tensile stress-strain measurements (b), Figure S2: (1) Photographs of homogeneous and transparent aqueous solutions, that may be used for further casting; (2,3) zinc and copper containing copolymers obtained by polymerization in ampoules. Figure S3: TGA curves of obtained copolymers (nitrogen, 10 K/min), Figure S4: DSC curves of obtained copolymers (nitrogen, 5 K/min) Figure S5: Photographs of the healing process of gel 5 demonstrating their mechanical deformation and recoverability: pristine sample (I), the sample has increased elasticity (II), cut of the sample (III), the sample after self-healing after 8 h (IV), after 48 h (V). Figure S6: Photographs of the healing process of gel 6: original sample (I), cut of the original sample (II), sample after self-healing after 8 h (III), after 48 h (IV), elasticity sample after healing (V).
