# Supplementary material for: Novel Self-Healing Metallocopolymers with Pendent 4-Phenyl-2,2′:6′,2″-terpyridine Ligand: Kinetic Studies and Mechanical Properties
Source: Polymers (Basel). 2021 May 27;13(11):1760. doi: 10.3390/polym13111760 (PMC8199432; doi:10.3390/polym13111760)
Supplement: Supplementary file 1 [file polymers-13-01760-s001.zip › polymers-1226917-supplementary.pdf]

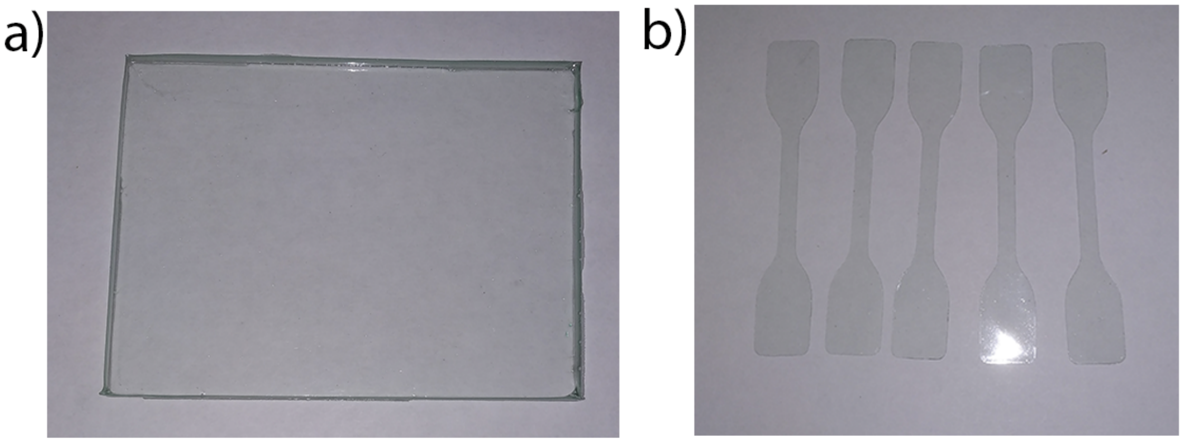

Figure S1. Gel sheet (a) and the samples for the tensile stress-strain measurements (b)

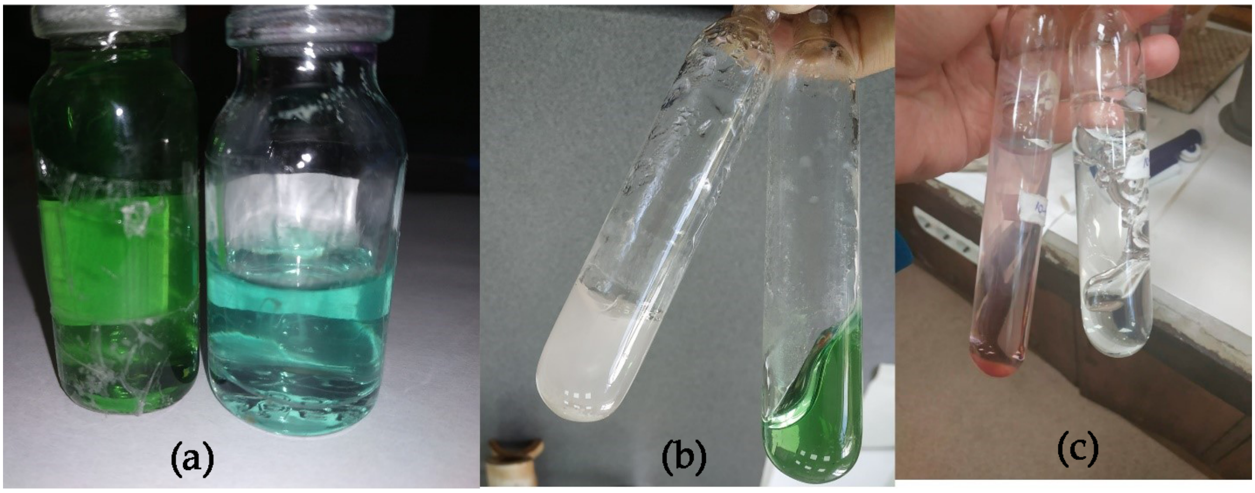

Figure S2. From left to right:a) homogeneous and transparent aqueous solutions that can be used for further casting; b,c) zinc- and copper-containing copolymers obtained by polymerization in ampoules.

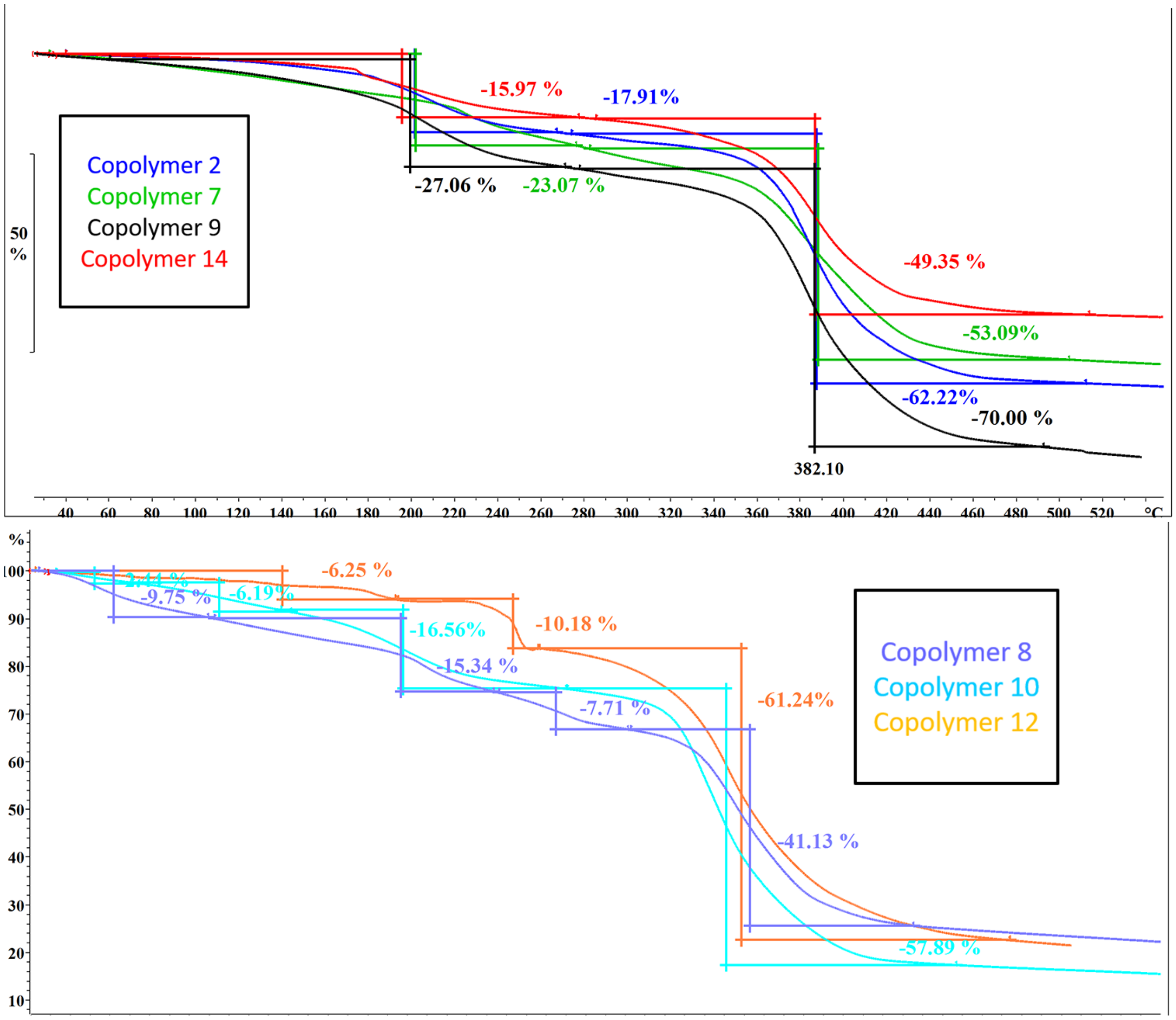

Figure S3. TGA curves of obtained copolymers (nitrogen, 10 K/min): Copolymer 2 – AM/AA = 50/50 (wt.%), copolymer 7 – AM/AA/MCM2 = 84.5/14.5/1 (wt.%), copolymer 9 – AM/AA/MCM2 = 49.5/49.5/1 (wt.%), copolymer 14 - 49.5/49.5/1 (wt.%), copolymer 8 – AM/AA/MCM1 = 84.5/14.5/1 (wt.%), copolymer 10 – AM/AA/MCM1 = 49.5/49.5/1 (wt.%), copolymer 12 – AM/AA/MCM1 = 14.5/84.5/1 1 (wt.%).

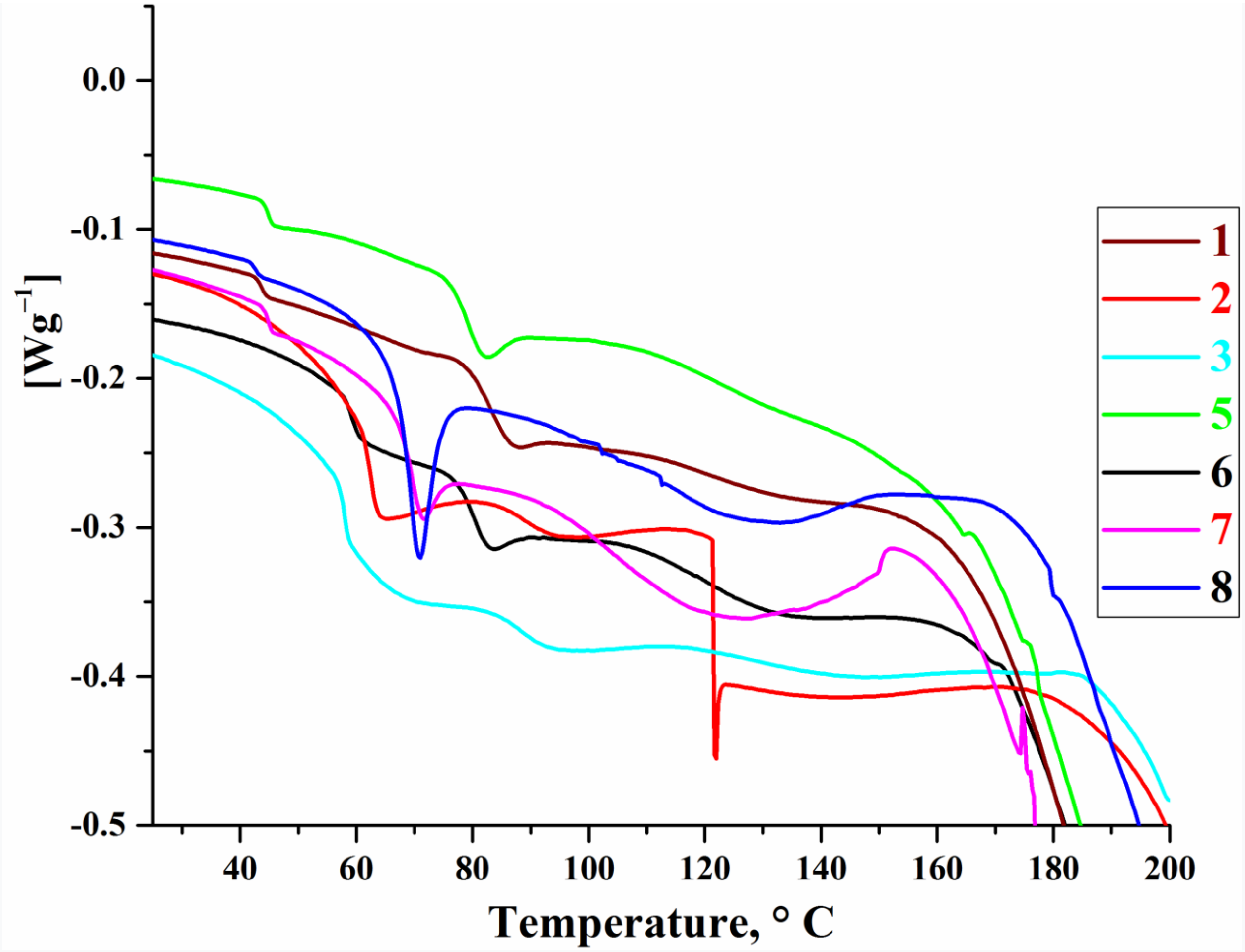

Figure S4. DSC curves of obtained copolymers (nitrogen, 5 K/min)/ Copolymers: 1 - AM/AA = 15/85 (wt.%), 2 – AM/AA = 50/50 (wt.%), 3 – AM/AA = 85/15 (wt.%), 5 – AM/AA/MCM1 = 84.5/14.25/1.25 (wt.%), 6 – AM/AA/MCM2 = 84.5/14.25/1.25 (wt.%), 7 – AM/AA/MCM2 = 84.5/14.5/1 (wt.%), 8 – AM/AA/MCM1 = 84.5/14.5/1 (wt.%).

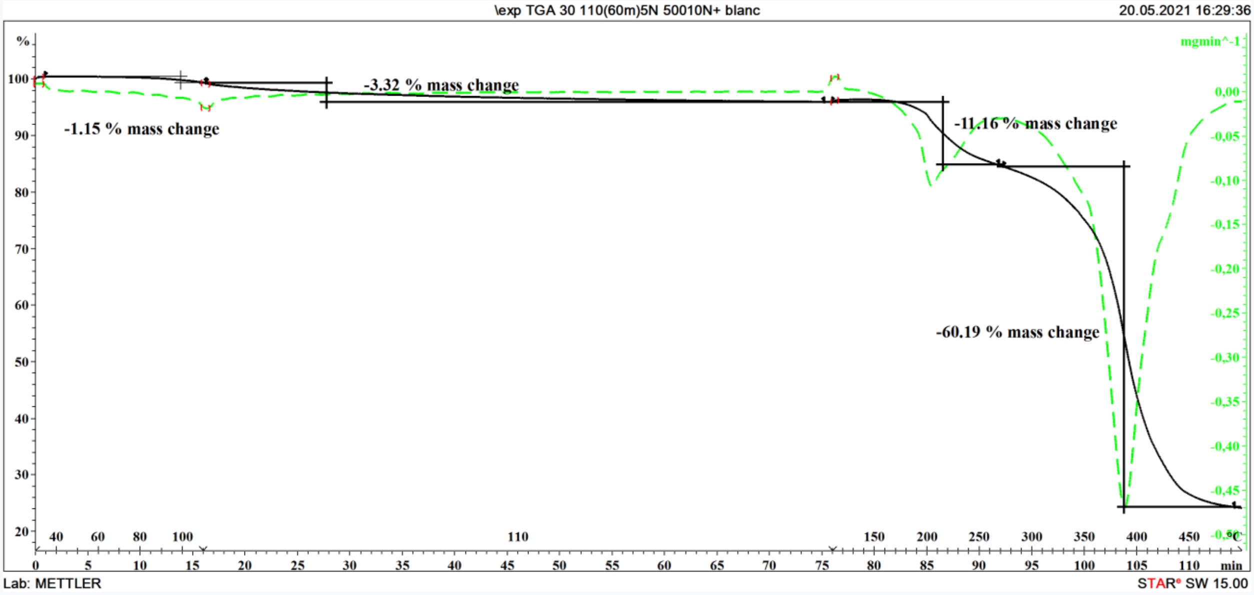

Figure S5. TGA curve of copolymer 14 [AM/AA/MCM1= 49.5/49.5/1 %/%/%], which has kept slightly above 100 °C for 1 hour

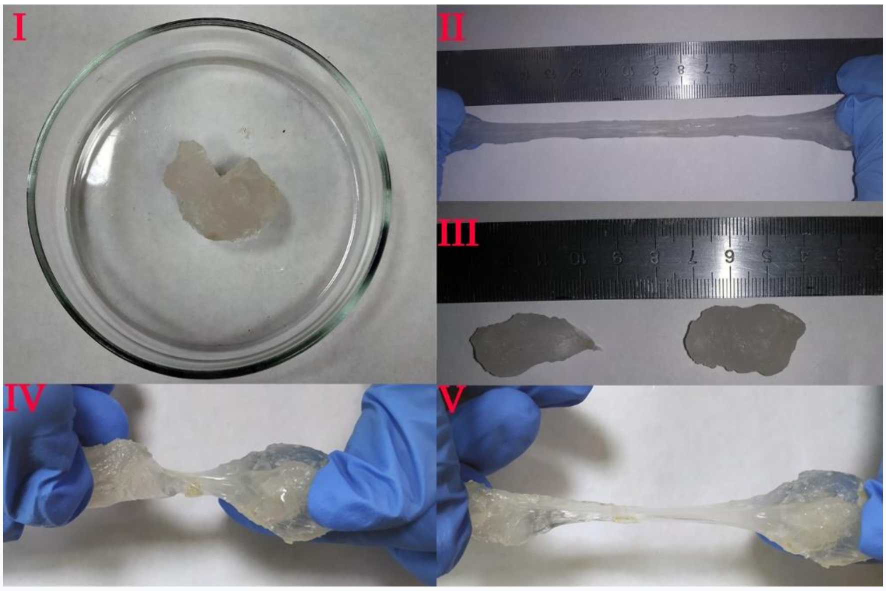

Figure S6. Photographs of the healing process of gel 5 (AM/AA/MCM1 = 84.5/14.25/1.25 (wt.%)) demonstrating their mechanical deformation and recoverability: pristine sample (I), the sample has increased elasticity (II), cut of the sample (III), the sample after self-healing after 8 (IV) and 48 hours (V).

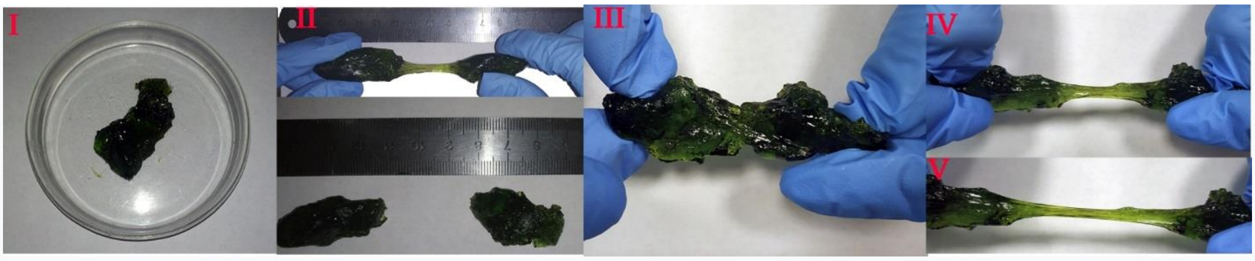

Figure S7. Photographs of the healing process of gel 6 (AM/AA/MCM2 = 84.5/14.25/1.25 (wt.%)): original sample (I), cut of the original sample (II), sample after self-healing after 8 (III) and 48 hours (IV), elasticity sample after healing (V).
